# SLEEP IN FOCUS: EVALUATING WEARABLE BIOMETRIC DEVICES FOR DEMENTIA SLEEP MONITORING- A SCOPING REVIEW OF EFFICACY

**DOI:** 10.1093/geroni/igae098.4076

**Published:** 2024-12-31

**Authors:** Leroy Griffiths, Ellen McCreedy, Ann Reddy

**Affiliations:** Brown University, Providence, Rhode Island, United States; Veterans’ Administration, Providence, Rhode Island, United States; Brown University School of Public Health, Providence, Rhode Island, United States

## Abstract

Sleep disturbances are prevalent among individuals with Alzheimer’s disease and related dementias (ADRD) and adversely affect their physical and cognitive functioning. Monitoring sleep patterns, therefore, is imperative for assessing cognitive deterioration. Wearable devices may provide a cost-effective, non-invasive approach for conducting sleep studies in people with ADRD, provided they are tolerable and accurate. We conducted a scoping review to evaluate the efficacy of wearable devices in measuring sleep among people with ADRD, compared to gold standard measures: polysomnography (PSG) or sleep diaries. Utilizing Pubmed, we identified 140 potentially relevant articles. Of these, 104 were excluded during abstract review, 33 during full-text review, and 3 met the inclusion criteria for analysis. The primary reasons for full-text review exclusion were: lack of a gold standard comparator or direct measure comparison (76%), incorrect study population (12%), and studies involving randomized controlled trials without baseline data (6%). The included studies indicated that actigraphy consistently overestimated total sleep time and sleep disturbances compared to sleep diaries and questionnaires. Results are consistent with previous research that suggests actigraphy may overestimate sleep in low-mobility populations. It may also indicate that sleep diaries are ineffective for this population. Since none of the studies utilized PSG, more research is necessary to establish efficacy. Similarly, if sleep diaries are imprecise, the effectiveness of wearables may be insufficiently substantiated. Furthermore, none of the included studies report sample characteristics regarding race, despite the optical technology utilized in wearables being linked to racial bias in individuals with darker skin tones.

